# Enhancing the Ag-loading capacity on Ti_3_C_2_T_*x*_ sheets as hybrid fillers to form composite coatings with excellent antibacterial properties†

**DOI:** 10.1039/d3ra05188a

**Published:** 2023-10-03

**Authors:** Yajun Deng, Zijie Zhou, Changan Zhang, Hui Li, Jianfeng Lan, Jianhua Wu, Shibin Wang

**Affiliations:** a Xiamen Key Laboratory of Marine Corrosion and Intelligent Protection Materials, Jimei University Xiamen 361021 China dengyj@jmu.edu.cn

## Abstract

The settlement of microorganisms is an unwanted process in various practical fields, where also the first attaching microorganisms could promote other bacterial adhesion, causing an acceleration of bioaccumulation on the solid surface and damage to the surface functions. Developing an advanced composite coating with anti-microorganism attachment features is still a big challenge, and the critical element in any such method is to find an efficient functional agent for use in the coating system that could extend the service period. MXenes have received increasing attentions owing to their unique layer structure and large specific surface area. Increasing studies have been devoted to the development of MXene/polymer composites with creatively designed structures to realize various specific functions. Herein, two-dimensional (2D) transition metal carbide material MXene as a carrier was etched and decorated with cellulose to enhance the anchor points to grasp functional Ag nanoparticles *via* a simple method. The MXene nanosheets (Ti_3_C_2_T_*x*_) were modified by cellulose to graft hydroxy groups on their surface, and then they were incorporated into silver nanoparticles (Ag NPs). The results showed that the cellulose could increase the loading content of the Ag NPs on the MXene surface, and also could act as a stabilized material to form the composite filler MXene@cellulose@Ag NPs (MAC), which could serve as a functional agent. Furthermore, the obtained product MAC filler exhibited excellent dispersibility and stability among all the tested fillers (MXene and MA), and it could help avoid aggregation and promote homogenous dispersal in the coating network. Besides, MAC displayed outstanding antibacterial activities against *E. coli* and *S. aureus* at the same concentration among all the fillers. When the filler was embedded into the coating system, the composite coating PCB-MAC possessed abundant active Ag^+^ ions released by the Ag NPs, which could work against bacterial growth and achieve a favorable antibacterial inhibition effect. Therefore, we believe that the active MAC filler maintained high antibacterial efficiency, evincing its potential as a desirable agent for obtaining an excellent anti-adhesive behavior in numerous broad applications, such as the environment field or medical area.

## Abstract 2

The settlement of microorganisms is an unwanted process in various practical fields, where also the first attaching microorganisms could promote other bacterial adhesion, causing an acceleration of bioaccumulation on the solid surface and damage to the surface functions.

## Graphical abstract

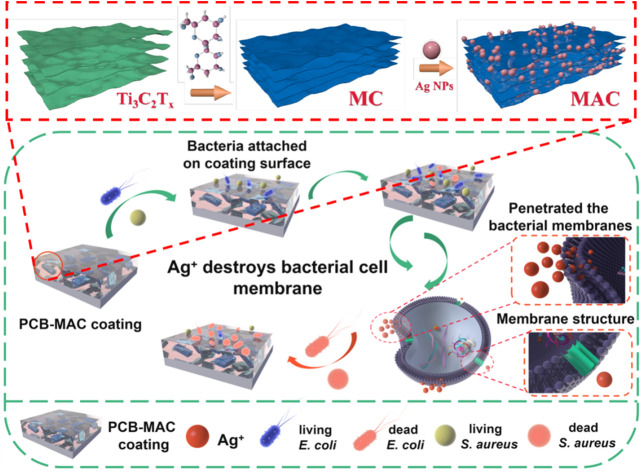

## 1. Introduction

Microorganism attachment is a common phenomenon in various application fields, including medicine, food, and even ocean engineering, resulting in potential safety hazards. Microorganism attachment is the preliminary behavior toward supporting a nutrient microenvironment that can facilitate other organisms to adhere, and bacteria growth is then ubiquitous and could easily generate a biofilm when attaching on a target surface. To confront this challenge, much effort has been devoted to exploring surface treatment or surface modification methods to achieve an improved bacterial resistance performance. One of the surface treatment methods is to cover the antibacterial film or coat the antibacterial organic material hybrids; for example, selecting the appropriate active additives to incorporate with an organic coating to form a composite.

MXene, which is a class of 2D transition metal carbide, has attracted considerable attentions since its discovery in 2011 by Gogotsi.^1–4^ MXenes are classically obtained *via* etching selectively an A layer from an M_*n*+1_AX_*n*_ precursor, where M represents the transition metal elements, A represents the group 3 and 4 elements, X denotes N or C, and *n* is generally 1 to 3. Therefore, the M_*n*+1_AX_*n*_ structure is formed of M_*n*+1_X_*n*_ layers and obtains the 2D dimensional structure M_*n*+1_X_*n*_T_*x*_, where T_*x*_ represents the terminal groups, such as –OH, –O, –F.^5–7^ Owing to their excellent properties, such as metallic conductivity, and mechanical and thermal stability, these materials are applied to reinforce a polymer matrix to enhance its hardness, strength, and the other functional properties in many intensive potential applications in a variety of areas, such as membrane, coating, photocatalysts.^8,9^ In particular, MXene is naturally hydrophilic and exhibits expedient decoration due to the terminal functional groups (–OH, –O, –F) on the surface, and the multilayer or few-layer structures are supporting a large specific surface area, suggesting it could be a promising carrier for advanced materials, and, particularly, a good candidate for preparing functional materials to potentially utilize in coating systems. However, MXene is unstable in humid environments and this disadvantage has obviously hindered its fabrication, storage, and even applications to date.^10^ With regard to this problem, various methods have been proposed and adopted, such as storage in an inert environment, adding a functional agent, like antioxidant, anticorrosion, or antifouling additives, and decorating the surface by certain molecules or ions to achieve a stable microstructure.^11^

With inherent broad-spectrum antibacterial properties, metal ions are the most common and traditionally active bactericidal agents.^12,13^ The conventional method to endow a coating with antibacterial performance is to introduce a suitable functional agent into the matrix, which could then develop the strong antibacterial properties. Silver nanoparticles (Ag NPs) are a kind of remarkable antibacterial agent. Therefore, adding Ag NPs onto the surface or into the interlayer of MXene could improve the antibacterial behavior and lengthen the durable antibacterial effect. Besides, the high specific surface area of MXene makes it a suitable template for immobilizing Ag NPs, which could address many problems of Ag NPs, such as their aggregation and cumulative toxicity, and could avoid the degradation of their active performances.^14–16^ With regard to the outstanding antibacterial behavior of Ag NPs, according to studies, the specific process of killing the bacteria is as following. First, Ag^+^ ions from the Ag NPs interfere with the bacterial cell membrane, attack the membrane, and disturb the transmembrane transport, leading to leakage of the cell content, deactivation of the functional proteins, and shrinkage of the genetic materials. Besides, Ag NPs can stimulate the formation of reactive oxygen species (ROS), which can affect the respiration enzymes to disturb the synthesis of ATP, lose the protein replication abilities, and eventually cause bacterial death.^17–21^

In a sense, the capacity to interfere in bacterial metabolism is closely related to the release of Ag^+^ ions, and so herein, the loading amounts of Ag NPs was considered as an important key to promoting the enhanced antibacterial effect of MXene composites. Recently, Ag–Ti_3_C_2_ nanohybrids were fabricated *via* an *in situ* reduction method and exhibited excellent photodegradation efficiency due to the good conductivity of Ti_3_C_2_ and Schottky barriers of the interfacial Ag–TiO_2−*x*_.^22^ This method revealed that the assistance of MXene could stabilize the Ag NPs in the hybrid system, and boost their efficient function in photocatalysis. Zhou *et al.* explored the antibacterial property of hierarchical MXene@Ag hybrids prepared in an *in situ* reduction way, and the results showed that the bacterial killing behavior of the composite epoxy resin could be improved effectively.^23^ Unfortunately, the *in situ* reduction of Ag NPs on the MXene surface led to some obvious disadvantages, including a durability deficiency, heterogeneous morphology, and a small amount loading on the MXene surface.

Herein, in this work, two-dimensional transition metal carbide material MXene with a large specific surface area was utilized as a carrier after decorating with cellulose. The cellulose was added to enhance the stability of MXene and to increase the anchor points to grasp the reinforced agents, *i.e.*, the Ag NPs. This method aimed to increase the loading contents of Ag NPs and to enhance the stability of the MXene@cellulose@Ag NPs (MAC) hybrid agent. Furthermore, on the basis of a previous work, polybenzoxazine resin PCB^24^ was chosen as the carrier for the different fillers to evaluate the antibacterial properties in the composite coating network. It is believed that the prepared active agent and the composite coating have great potential for applications in medical equipment or for antibacterial surfaces.

## 2. Experimental section

### 2.1 Materials

Ti_3_AlC_2_ powder and silver nitrate (AgNO_3_) were provided by Macklin Co., Ltd. Polyethyleneimine (PEI, *M*_w_ = 10 000 g mol^−1^), lithium fluoride (LiF), hydrochloric acid (HCl), acetone, and ethyl alcohol were purchased from Sinopharm Chemical Reagent Co., Ltd. Ethylene glycol (EG), polyvinylpyrrolidone (PVP), and cellulose were obtained from Sigma-Aldrich. All the commercial chemicals were used as received.

### 2.2 Preparation of the Ti_3_C_2_T_*x*_ and functional Ti_3_C_2_T_*x*_ sheets

Ti_3_C_2_T_*x*_ sheets were modified according to a previous method through the following steps:^25^ (1) 1 g Ti_3_AlC_2_ powders as the precursor were added into an etchant solution containing 12 M LiF and 9 M HCl, and then the mixture was continuously stirred at room temperature for 24 h; (2) the obtained mixture was washed with deionized water and transferred to a centrifuge (3500 rpm) to remove the residual acid until the solvent pH value reached 6–7; (3) the Ti_3_C_2_T_*x*_ sheets were ultrasonically dispersed in deionized water and further centrifuged to obtain a solid, and finally Ti_3_C_2_T_*x*_ powders were obtained after 48 h freeze-drying.

Next, 100 mg of the as-obtained Ti_3_C_2_T_*x*_ powders and 400 mg PEI were ultrasonically dispersed in 200 mL deionized water, and vigorously stirred for 24 h at room temperature. The mixture was washed with deionized water by centrifugation (3500 rpm) to obtain a solid. Finally, the modified powders (M-PEI) were collected after 48 h vacuum freeze-drying at −80 °C.

### 2.3 Fabrication of the MA and MAC fillers

The silver nanoparticles were fabricated by the following reported steps:^26^ (1) silver nitrate (AgNO_3_, 1.0 g), polyvinylpyrrolidone (PVP, 5.0 g), and ethylene glycol (EG, 500 mL) were added into a three-necked flask, and the mixture was continuously stirred at 130 °C for 2 h; (2) after 2 h stirring, the mixture was heated at 130 °C for 8 h without any stirring. Acetone was then added into the obtained mixture, and stirred with ultrasonication for 5 min. Afterwards, the mixture was separated by centrifugation, washed by ethanol three times, and dried at 60 °C for 24 h to obtain the Ag NPs.

M-PEI powders (0.4 g), Ag NPs (0.6 g), and ethanol (200 mL) were added into 250 mL flask. The mixture was stirred at 30 °C for 60 min, and then the mixture was separated by centrifugation at 8000 rpm for 10 min, washed by ethanol twice, and collected by vacuum freeze-drying after 48 h at −80 °C. The powder was MXene@Ag nanocomposites (MA). The Ag NPs loading amount of the MA filler was about 24.1 wt% of the total mass fraction, as shown in Fig. S1 and S2.†

Cellulose (0.8 g) was mixed into ethanol (100 mL) and stirred for 3 min to obtain a homogenously dispersed solution. Then, 0.4 g M-PEI powders was added into the above solution and stirred for 30 min. After that, 0.6 g Ag NPs was added into the aforementioned mixture and then continuously stirred for 24 h. Finally, the mixture was centrifugated at 8000 rpm for 10 min, washed by ethanol twice, and vacuum freeze-dried for 48 h. The powder was MXene@cellulose@Ag nanocomposites (MAC). The Ag NPs loading amount of MAC filler was about 47.6 wt% of the total mass fraction, as shown in Fig. S3 and S4.†

### 2.4 Preparation of the composite polybenzoxazine resins

The composite polybenzoxazine resin was generally fabricated as follows: (1) benzoxazine monomer was prepared according to a previous work.^24^ Specifically, curcumin (0.01 mol, 3.68 g), paraformaldehyde (0.04 mol, 1.20 g), and 3-aminopropyltriethoxysilane (APTES, 0.02 mol, 4.42 g) were added into a 250 mL flask filled with chloroform (100 mL) followed by stirring and refluxing for about 48 h at 96 °C. After the reaction had finished, the mixture was allowed to cool down and the product was separated and purified to obtain the pure benzoxazine monomer (CB). Then, the monomer was dissolved in tetrahydrofuran to form a homogeneous solution; (2) the nanocomposites, including MXene, MA, and MAC, were respectively dispersed in tetrahydrofuran, and the mixtures were ultrasonicated for 10 min, respectively; (3) the nanocomposite dispersions were separately added into the monomer homogeneous solution, and the mixtures were magnetically stirred for 8 h at 45 °C separately; (4) the redundant solvent was removed from the mixture *via* a rotary evaporator, and the concentrated mixture containing 5 wt% various nanocomposites was spin-coated onto cleaned glass plates (25 mm × 25 mm × 1.5 mm), which were respectively washed by acetone and ethanol; (5) all the coated samples were cured at temperatures from 60 to 200 °C at a heating rate of 20 °C/20 min, and then the samples were heated at 200 °C for 1 h. The coating containing the MXene, MA, and MAC nanocomposite were named as PCB-M, PCB-MA, and PCB-MAC, respectively. The PCB coating was prepared following the same procedures except for the addition of any nanocomposite. All the coating thicknesses were about 25 ± 5 μm.

### 2.5 Characterization

The Fourier transform infrared (FT-IR) spectra of the samples were conducted on a Nicolet iS50 spectrometer in the range from 4000 to 500 cm^−1^. Surface morphology analysis of the nanoparticles was performed by scanning electron microscopy (SEM, Hitachi FE-SEM 4800, Japan) observation under 10 kV. The Raman spectra of the different nanoparticles were recorded on a confocal Raman spectrometer (LabRAM HR Evolution, Horiba, Japan) utilizing an excitation wavelength of 532 nm, with LabSpec6 software used for to process the Raman spectra. Thermogravimetric analysis (TGA, Netzsch STA 449F3 Jupiter, Germany) was performed at a heating rate of 10 °C min^−1^ under a N_2_ flow with a 50 mL min^−1^ flow rate in the temperature range of 25–800 °C. The micromorphology of the nanoparticles was observed by transmission electron microscopy (TEM, Talos F200s, USA) under an acceleration voltage of 15 kV. Also, X-ray diffraction (XRD, D8-A25, Bruker axs) was performed to investigate the crystalline structure of the samples.

#### 2.5.1. Antibacterial assessment

The assay was performed according to a previous method.^27^ Specifically, *Staphylococcus aureus* (*S. aureus*, ATCC 6358P) and *Escherichia coli* (*E. coli*, DH5α), as the representative Gram-positive and Gram-negative bacteria are typically employed as indicators to judge the antibacterial activity. Various concentrations of the different nanotubes (including MXene, MA, and MAC at 0, 4, 12, 20, and 24 mg mL^−1^) were charged into *S. aureus* or *E. coli* bacterial suspensions (1 mL, 1 × 10^7^ cfus mL^−1^) in conical flasks. The mixtures containing the nanotubes and the bacterial suspension were respectively incubated at 120 rpm in a shaking incubator at 37 °C for 12 h. Afterwards, 100 μL aliquots of the bacterial suspensions were respectively extracted and added into the Luria–Bertani agar plate, and then the plates were placed in the incubator at 37 °C for 24 h. Images of the bacteria were recorded. Each of the experiments was repeated at least three times.

#### 2.5.2. Bacterial anti-adhesion of the different coatings

The antifouling performance of the coatings was evaluated according to a method in a previous work.^28^ Specifically, the various coatings (10 mm × 10 mm × 1.5 mm) were respectively added into the bacterial suspensions (*S. aureus* and *E. coli*, 1 × 10^7^ cfus mL^−1^) in a 24-well plate, and the hybrid suspensions were cultured for 6 h at 37 °C under 120 rpm shaking. Afterwards, the bacterial suspensions were respectively extracted and the coatings were rinsed in sterilized phosphate buffer saline (PBS) solution to remove the unattached bacteria, and then Syto-9 (2 mg mL^−1^) was added into the plate to stain the samples at 23 °C for 15 min in the dark condition. Finally, the bacteria on the various sample surfaces were observed using an Olympus IX73 fluorescence microscope.

#### 2.5.3. SEM observation

The morphologies of the bacteria (*S. aureus* or *E. coli*) on the coating surfaces were separately observed and detected by SEM after the bacterial adhesive resistance test.^24^ Specifically, the bacteria attached on the coating surface were rinsed by sterilized PBS twice, fixed with 2.5 wt% glutaraldehyde solution for 4 h, and then dehydrated for 10 min twice sequentially with 30%, 50%, 75%, 90%, and 100% ethanol. Finally, the morphologies of the dried bacterial on the coating surface were observed and recorded by SEM.

## 3. Results and discussion

This study focused on increasing the Ag NPs loading content on the surface of MXene by a novel and simple method. Through the greater loading of Ag NPs, while avoiding the aggregation of the Ag NPs, the antibacterial behavior of MXene could be improved. This represents a meaningful and promising approach to endowing the coating material with specific functions and to meeting the demands of the colloid surface in specific applications.

### 3.1 Morphologies and compositions of the microstructures

**Fig. 1:**
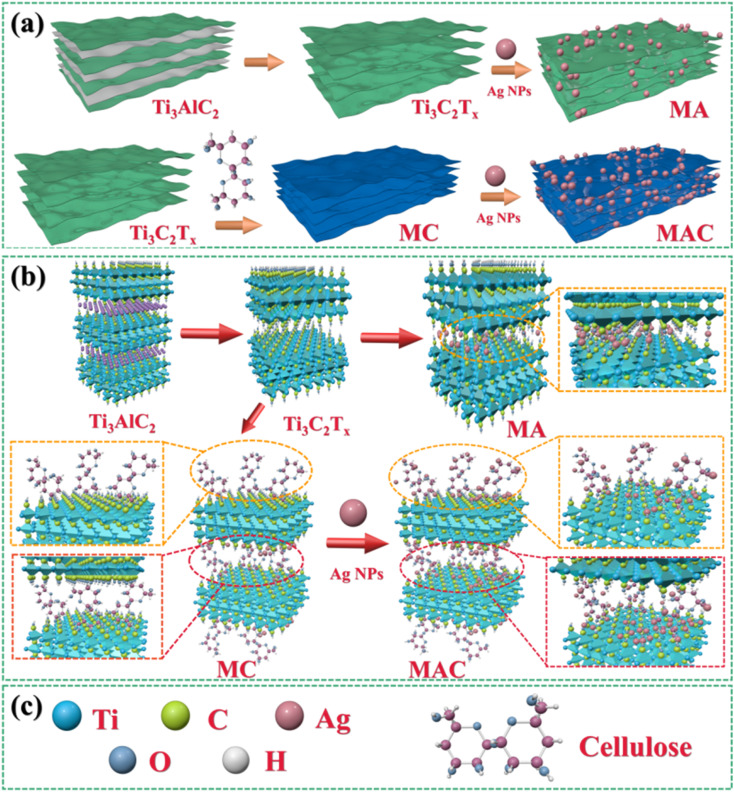
. Synthesis methods and fabrication processes (a and b) for obtaining Ti_3_C_2_T_*x*_ (MXene) by Ti_3_AlC_2_, MA, MC, and MAC;^30^ (a–c) schematic illustration of the molecular model for Ti_3_C_2_T_*x*_ (MXene), MA, MC and MAC.

The synthesized MXene samples were prepared through a “top-down” method by using HF wet-etching to remove one or more atomic layers from the Ti_3_AlC_2_ phase to form the designed Ti_3_C_2_T_*x*_. Herein, when the Al layer is etched away, the Ti_3_C_2_ naturally forms –O, –F, –OH, where T represents the terminating surface groups in the Ti_3_C_2_T_*x*_ structure,^29^ as shown in Fig. 1a. Then, the Ag nanoparticles (Ag NPs) were modified on the definite surface layer thanks to the addition of PVP and PEG,^28^ and this layer was identified by the hydroxyl groups, which were utilized as anchors to hold the terminating sites on the Ti_3_C_2_T_*x*_ sheets. Then, Ag NPs were directly loaded on the Ti_3_C_2_T_*x*_ sheets for comparison, and this samples was named as the MA hybrid. To vividly demonstrate the three-dimensional structure, a schematic illustration of MA is provided in Fig. 1a. Furthermore, for increasing the anchors to hold more Ag NPs on the Ti_3_C_2_T_*x*_ layer, one of the promising methods is to decorate the Ti_3_C_2_T_*x*_ surface to meet the needs for higher loading concentrations. Cellulose was used here as a natural material that possessed the required hydroxyl groups to graft on the Ti_3_C_2_T_*x*_ surface (as MC in Fig. 1b) and to enhance the holding sites to grasp more Ag NPs to form the target product MAC, as shown in Fig. 1a and b. Besides, the modification of MXene by cellulose could avoid the Ag NPs aggregation and enhance their dispersibility *via* the hydrogen or covalent bonds of the molecular links.

**Fig. 2:**
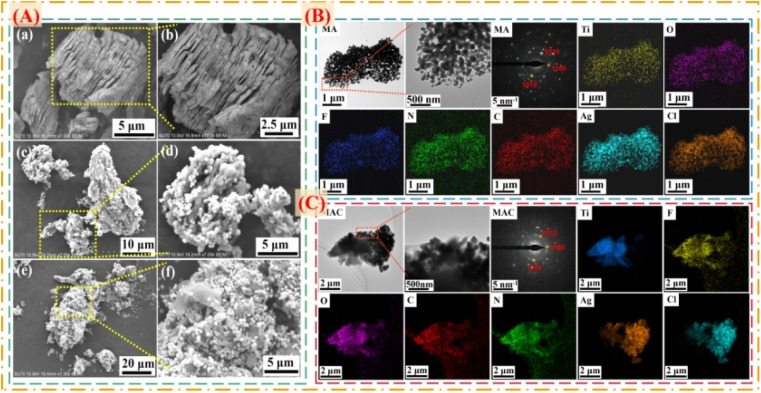
(A) SEM images of the composite fillers of (a and b) MXene, (c and d) MA, and (e and f) MAC. TEM, SAED, and elemental mapping images of (B) MA and (C) MAC.

To prove the surface morphology and the structure composites of the different hybrids, SEM and TEM were used to accurately analyze the structural morphologies and composite elements of the MA and MAC hybrids, as shown in Fig. 2. As shown in Fig. 2A, the Al layer was removed from the Ti_3_AlC_2_ sheet to form the multilayer structure Ti_3_C_2_T_*x*_ (MXene) after sufficient HF etching (Fig. 2a and b). The unique surface and interface engineering of MXene could provide a huge surface area containing functional groups such as –O, –OH, and –F, which could act as the sites to load the Ag NPs on the MXene layer *via* hydrogen bonding, van der Waals forces, and hydrogen bonding (Fig. 2c and d). However, the Ag NPs led to a lower loading *via* straightforward attachment on the MXene surface according to the images in Fig. 2c and d. The surface modification or functionalization of the carrier MXene has attracted much interest for directly quantifying nanomaterial species. Thus, by modifying the surface with cellulose as grafted-on chains, the MXene sheets can gain increased functional sites to anchor more Ag NPs, as shown in Fig. 2e and f. To confirm this, the following tests were done focused on the elements in the different fillers, including MXene, MA, and MAC. The TEM morphologies and element mappings of MA and MAC are presented in Fig. 2B and C, where it can be seen that MA and MAC showed dense Ag NPs on the MXene surface with the presence of clear diffraction sites of the (01̄10), (11̄00), and (101̄0) facets, indicating the existence of MXene.^31,32^ Also, the element mapping images demonstrated the uniform distributions of Ti, O, F, N, C, Ag, and Cl. As above-mentioned, the MXene modified by cellulose displayed a greater loading concentration of Ag NPs, obtained *via* a simple approach to achieve the desirable structure for endowing it with antibacterial properties, and thus making it a potential candidate for larger-scale use in future biological surface applications.

### 3.2 Characterization and analysis

**Fig. 3:**
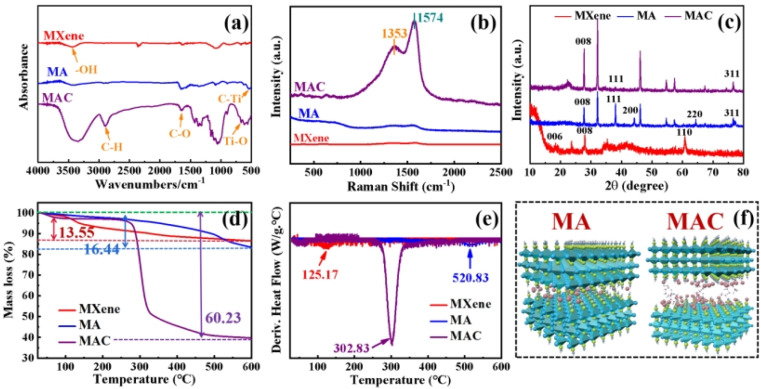
(a) FT-IR spectra of the different composites (MXene, MA, and MAC); (b) Raman spectra of the different composites; (c) XRD patterns of the different composites; (d) TG and (e) DTA curves of the different composites; and (f) molecular models of MA and MAC.

In order to clarify the structural characterization, the chemical compositions of MXene, and the hybrids MA and MAC were measured by FT-IR, Raman, and XRD analysis. As shown in Fig. 3a, from FT-IR spectra, intense and broad absorption band ranges at 3277–3440 cm^−1^ could be observed, belonging to the –OH group, which was assigned to the combination of –OH on the Ti_3_C_2_T_*x*_ nanosheets and the Ag NPs surface. Other peaks that appeared at 674 and 557 cm^−1^ corresponded to Ti–O and C–Ti characteristic vibrations.^33^ Furthermore, absorption peaks at 2893 and 1630 cm^−1^ were noted and assigned to the C–H and C–O bending vibrations.^23^ It was noted that the C–H and C–O groups were decorated on the surfaces of the Ag NPs and provided specific sites to anchor on the MXene nanosheets.

Besides, as shown in Fig. 3b in the Raman spectra, absorption peaks were observed at 1353 and 1574 cm^−1^, which were attributed to C–N and C=C groups of the different nanocomposites and caused by the remaining surfactants or the reducing agents from oleic acid, oleylamine, or ascorbic acid on the Ag NPs. The absorption peaks of MAC were stronger than those of the MA owing to the grafted cellulose on the MXene surface.^34^ To further prove the successful preparation of MXene, and hybrids MA and MAC, the XRD patterns of the various samples were obtained and are shown in Fig. 3c. It could be seen that the characteristic peaks appeared at 28.0° and 61.0°, which corresponded to the (008) and (110) crystal faces of MXene.^23,35^ The peaks at 38.2°, 44.4°, 64.54°, and 77.0° respectively belonged to the (111), (200), (220), and (311) crystal faces of Ag NPs, and were close to the reported values,^36,37^ indicating the enrichment of the crystallinity of Ag NPs with the increasing concentrations of nanoparticles. Due to the higher loading concentrations of Ag NPs on the MXene surface, the specified peaks for MAC were stronger than those of the MA hybrid. This was due to the functional groups of cellulose on the MXene surface, which acted as the crucial sites to hold the dense Ag NPs, and these results were consistent with the morphologies obtained by the SEM and TEM analysis. All the aforementioned analyses demonstrated the successful preparation of the composites MA and MAC.

Thermogravimetric analysis was next performed and showed that the different fillers MXene, MA, and MAC exhibited decreased thermal stability. MXene was most thermally stable, with an 86.47% char yield, which was higher than that of the hybrids MA and MAC with 83.38% and 39.71% char yields, respectively. Because of the organic units of the Ag NPs surface on the MXene, MA showed less stability and its maximal weight loss was at 520.83 °C, as shown in Fig. 3d, e, and Table 1. Utilizing cellulose as the grafted-on chains to modify MXene, the MAC composite developed the lowest char yield of 39.71%, and showed the maximal weight loss at 302.83 °C, owing to the degradation of the grafted cellulose on the MXene surface and/or the organic units on the Ag NPs surface. From the above characterization results, the MA and MAC hybrids were further identified by their structure, and the desired molecular models could thus be described, as shown in Fig. 3f, to explain their morphologies and microstructures.

**Table 1:** Thermal parameters from the TG and TGA curves of the different fillers

Sample	*T*_5%_ (°C)	*T*_10%_ (°C)	*T*_max%_ (°C)	Char yield (%)
MXene	137.67	328.17	125.17	86.47
MA	352.83	492.33	520.83	83.38
MAC	275.00	288.17	302.83	39.71

### 3.3 Static settlement and stability of the different fillers

**Fig. 4:**
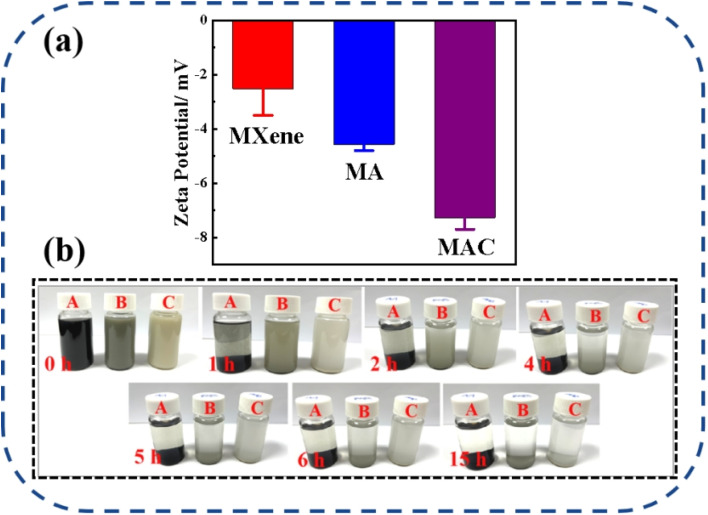
(a) Zeta potentials of the different fillers (MXene, MA, and MAC) in ethanol dispersion, (b) images of the different fillers with their static settlement, where A = MXene, B = MA, and C = MAC.

The dispersion stability of the different fillers, including MXene, MA, and MAC, was studied and is shown in Fig. 4. The zeta potentials of MXene, MA, and MAC with negative charges were respectively around −2.52 ± 0.98, −4.57 ± 0.23, and −7.27 ± 0.43 mV, as shown in Fig. 4a. The negative zeta potential value of MXene showed a higher negative charge owing to their aggregation in the ethanol dispersion. Due to the uniform structure of hydroxy groups on the Ag NPs surface, the MA dispersion showed a lower potential value than MXene, indicating the MA dispersion was more stable than that of MXene. Besides, the MAC dispersion displayed the lowest potential value. These results implied that cellulose served as a stabilizing agent for the MAC and could effectively avoid the Ag NPs aggregation and greatly enhance the dispersibility of the as-prepared solution. Actually, the optical photograph images in Fig. 4b of the different fillers could directly show the dispersion stability. As with the experimental results, the different fillers in ethanol solutions were homogeneous and uniform, with MXene exhibiting rapid sedimentation, causing the solid–liquid separation after 1 hour settlement. After 5 h settlement, MA showed a clear separation of the solid–liquid phase and a solid aggregation at the bottom of the bottle. MAC displayed the most stability compared to the MXene and MA fillers, and it exhibited agglomeration after 15 hours settlement. This phenomenon of the fillers was the same as shown in the zeta potential analysis. All the observations further confirmed that the target filler had been obtained, and the good stability of the MAC mixture could promote the compatibility between the coating and active filler to achieve the composite coating stabilization for the solid surface of medical devices.

### 3.4 Antibacterial and anti-adhesive performances

**Fig. 5:**
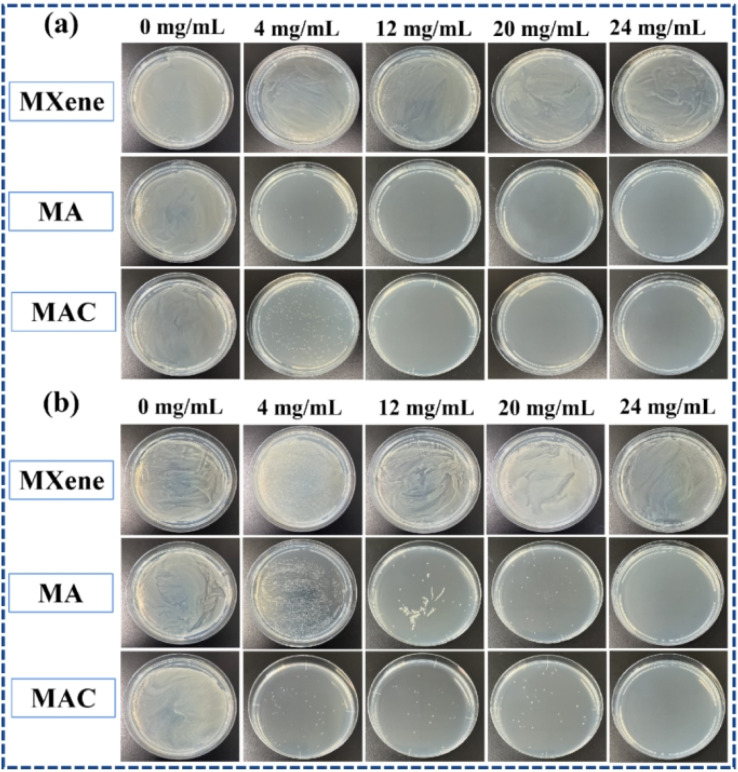
Bacterial plate photos of (a) *E. coli* and (b) *S. aureus* incubated with different concentrations of the composite fillers of MXene, MA, and MAC for 6 h incubation with 0 (control), 4, 12, 20, and 24 mg mL^−1^ concentrations, respectively.

As noted, biofouling is a negative impact and serious challenge to the protective coating used in many applications. Nanotechnology and the use of a polymer composite are two efficient methods to satisfy the need for a colloid surface to improve the antibacterial properties. The use of an active antibacterial agent is a directly valid method to enhance the functionalization of a coating. Herein, bacteria *E. coli* and *S. aureus* were used as indicators to evaluate the bacterial killing behavior of the agents. To probe the exact concentration needed to inhibit the growth of these two kinds of bacteria, different concentrations of the active agents, such as 0, 4, 12, 20, and 24 mg mL^−1^, were respectively added into the bacterial suspensions, and those suspension were extracted and added on to a Luria–Bertani agar plate to culture in the incubator. After 6 h incubation, the colonies of both *E. coli* and *S. aureus* were recorded to assess their growth situation. As shown in Fig. 5a and b, the comprehensive optimum concentration of the agent was 20 mg mL^−1^, which was found could best disturb the bacterial growth and reproduction, while MXene showed low antibacterial activity even at the highest concentration (24 mg mL^−1^). On the one hand, MXene exhibited some antibacterial capacity because it has sharp edges, which could destroy the adsorbed bacterial cell membrane *via* oxidation and physical processes.^38^ Besides, the abundant surface groups on the MXene surface when connecting with the bacterial membrane could promote the loss of bacterial nutrients and deactivate the cytoplasm of the cells through hydrogen bonding.^39^ On the other hand, relative to the Ag NPs, MXene showed an obviously weak antibacterial capacity. Thus, the synergistic effect of the Ag NPs to form a composite agent is an effective and important method for enhancing the antibacterial activity of MXene.

**Fig. 6:**
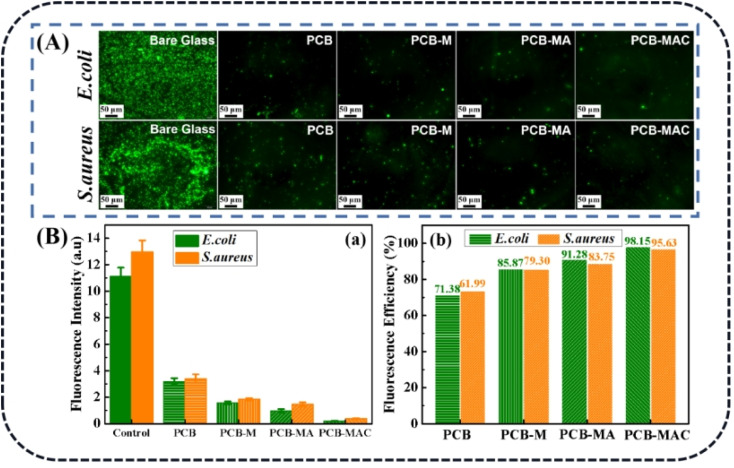
(A) Fluorescence microscopic images of the bacteria *E. coli* and *S. aureus* settled on different surfaces after 6 h of immersion in bacterial suspensions, (B)(a) fluorescence intensities and (B)(b) fluorescence efficiencies relative to bare glass of *E. coli* and *S. aureus* settled on different surfaces.

As for the aforementioned optimum antimicrobial concentration, we choose the agent concentration at 20 mg mL^−1^ to embed into the benzoxazine monomer to form the composite mixture and heat-cured it to give the crosslinked hybrid resins. Different resins containing the agents MXene, MA, and MAC were obtained and respectively named as PCB-M, PCB-MA, and PCB-MAC. The bacteria *E. coli* and *S. aureus* settlement on the different coatings surface was assessed, as shown in Fig. 6A, and the fluorescence intensity and efficiency of the stained coating surfaces were compared, as shown in Fig. 6B(a) and (b). Compared to the results for the fluorescence intensity of bare glass, the coatings including the PCB and the composite coatings (PCB-M, PCB-MA, and PCB-MAC) showed less bacterial attachment on the staining surface, and the fluorescence intensity of the PCB-MAC coating observed on the surface was the lowest brightness, compared to that of the control bare glass and other composite coatings. These results demonstrated that PCB-MAC exhibited a better antifouling activity against the bacteria, and the highest fluorescence efficiency.

**Fig. 7:**
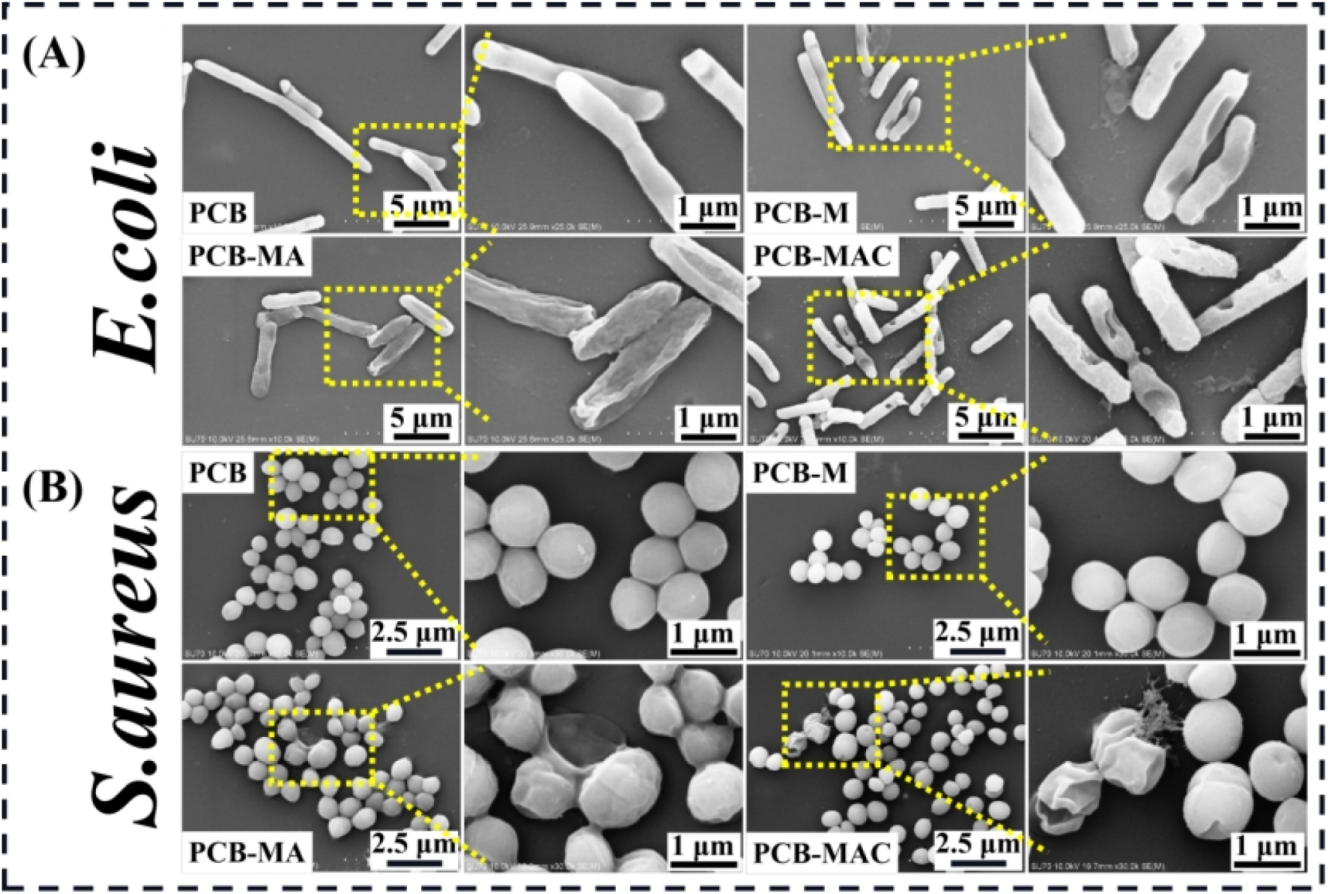
SEM microscopic images of the bacteria (A) *E. coli* and (B) *S. aureus* attached on the surfaces of the coatings PCB, PCB-M, PCB-MA, and PCB-MAC.

To clearly explore the growth states of the bacteria and the killing results by the active agents, the completed condition of the bacteria on the coating surface were observed to visualize the damaged morphologies of the bacterial membrane *via* SEM measurement, and the results are shown in Fig. 7A and B. PCB was used and tested as the control coating. It was found that *E. coli* seeded on the PCB surface with an intact rod shape, while the bacteria on the PCB-M coating surface had obviously shrunk and even caused holes to appear. Seriously, for the bacteria on the PCB-MAC coating, their membrane became rougher and more damaged than those on the PCB-MA surface. It could be clearly observed that most portions of the bacteria on the PCB-MAC coating were even destroyed. Likewise, *S. aureus* on the PCB and PCB-M coating surfaces exhibited a spherical shape with integrated and smooth membranes, while those bacteria seeded on the PCB-MA and PCB-MAC were seriously shrunk and showed damaged cells, which permeated with obvious lysis from the cell. The PCB-MAC coating revealed the best antibacterial behavior caused by the slow release of Ag^+^ ions, which could then attack the cell membrane and disturb the transmembrane transportation, thus damaging the bacterial metabolism.

**Fig. 8:**
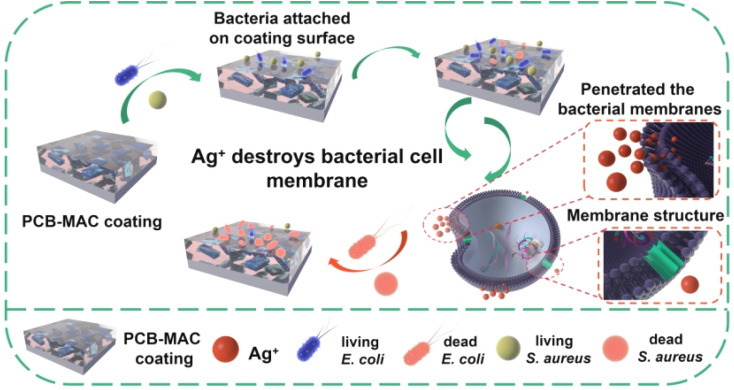
Schematic diagram of the antibacterial mechanism of the Ag nanoparticles.

A detailed schematic diagram of the bacterial killing activity of the PCB-MAC coating is shown in Fig. 8, where the significant antibacterial property of this coating was derived from the active filler MAC. Silver, one of the most well-known antibacterial agents, has been widely utilized to enhance the biocidal activity of materials, including the MXene sheet here. A prior interesting research reported that Ag/Ti_3_C_2_T_*x*_ was a better synergistic antibacterial model combining MXene with Ag^+^ release under special NIR irradiation, relative to Ag NPs or MXene alone.^40^ The composite antibacterial action has two keys factors. On the one hand, MXene enabled a larger surface area and improved sharp edges of the two-dimensional sheets, which could promote a better antibacterial property. Besides, the negative surface charges on the MXene surface were less effective against *E. coli*, which is a Gram-negative bacteria and can form a higher resistance to MXene sheets.^41,42^ On another hand, Ag NPs exhibited excellent antibacterial activity. The protons and the dissolved oxygen reduce the production of hydrogen oxides that would otherwise accelerate the oxidation of Ag to form unstable Ag^+^ ions.^19,20,43–45^ The Ag^+^ species from the Ag NPs reservoir could penetrate the bacterial cell membranes containing an outer membrane and inner plasma membrane to disturb the transmembrane transport. Thus, the Ag^+^ ions stimulate reactive oxygen species (ROS), which is a natural by-product of oxygenic respiration.^46^ The cell then endures high oxidative stress, leading to a cellular inactivation,^47,48^ including from the ROS attacking the protein to denaturalize physiological functions, and hinder the replication of DNA and mRNA, as well as damaging the bacterial metabolism. The outstanding sterilizing effect is thus related to the higher release concentration of Ag^+^ ions. Herein, the MAC agent showed obvious advantages because it utilized the MXene as a carrier to load more Ag NPs to avoid the aggregation of nanoparticles, and it could thus display excellent antibacterial activity at the same agent concentration when compared with the other agents in this work.

Based on the practical applications of MXene-based materials, these materials would need to take into consideration the possible toxicities. There are several comprehensive research reports on the evaluation of MXene composite agents. MXene can cause some cell cytotoxicity, attributed to the oxidative stress induced by the generated ROS,^49,50^ and the antibacterial action mechanism is the same as for Ag NPs. However, the cytotoxicity of Ag NPs is lower than that of Ag^+^, and the toxicity of Ag NPs is closely related to the structure, crystal defects, *etc.*^51^ ROS induction is a vital cause of the cytotoxicity of Ag NPs, and it also is the key product that can interfere with or inhibit the growth of bacteria. In this work, the composite fillers MA or MAC were slightly embedded into the coating system, and the coating network acted as the reservoir to release Ag^+^ from the Ag NPs on the solid surface. It could be speculated that there were few Ag^+^ enriched on the coating surface when the target composite coating was utilized as a protection for the solid surface, but the composite coating still developed an excellent antibacterial property, as shown in the above-mentioned results and as shown in Fig. 6.

## 4. Conclusion

In summary, we fabricated a simple and effective strategy to construct stable a 3D Ti_3_C_2_T_*x*_ MXene@cellulose@Ag (MAC) composite *via* the process of a direct grafting, precise capture, integration with anchors, and tailored loading for boosting the storage of Ag NPs on the MXene surface. Owing to the synergistic effect of the modified MXene as a carrier and Ag as the target load to achieve the expected functions, the MAC composite filler could serve as a potential antibacterial material in the antifouling coating field thanks to its higher loading capability, better stability, and high antibacterial performance. These properties could promote the compatibility between MAC and the coating network, and avoid Ag NPs aggregation in the coating system. This work proposes a simple method for the fabrication of a MXene composite filler, and gives a deep insight into different dimensional nanofillers to be assessed to develop synergistic effects for adjusting the antibacterial attachment property of polybenzoxazine coating.

## Conflicts of interest

There are no conflicts to declare.

## Supplementary Material

RA-013-D3RA05188A-s001
