# Supplementary material for: Enhancing the Ag-loading capacity on Ti3C2Tx sheets as hybrid fillers to form composite coatings with excellent antibacterial properties
Source: RSC Adv. 2023 Oct 3;13(41):28951–63. doi: 10.1039/d3ra05188a (PMC10545980; doi:10.1039/d3ra05188a)
Supplement: RA-013-D3RA05188A-s001 [file RA-013-D3RA05188A-s001.pdf]

Electronic Supplementary Material (ESI) for RSC Advances.
This journal is © The Royal Society of Chemistry 2023

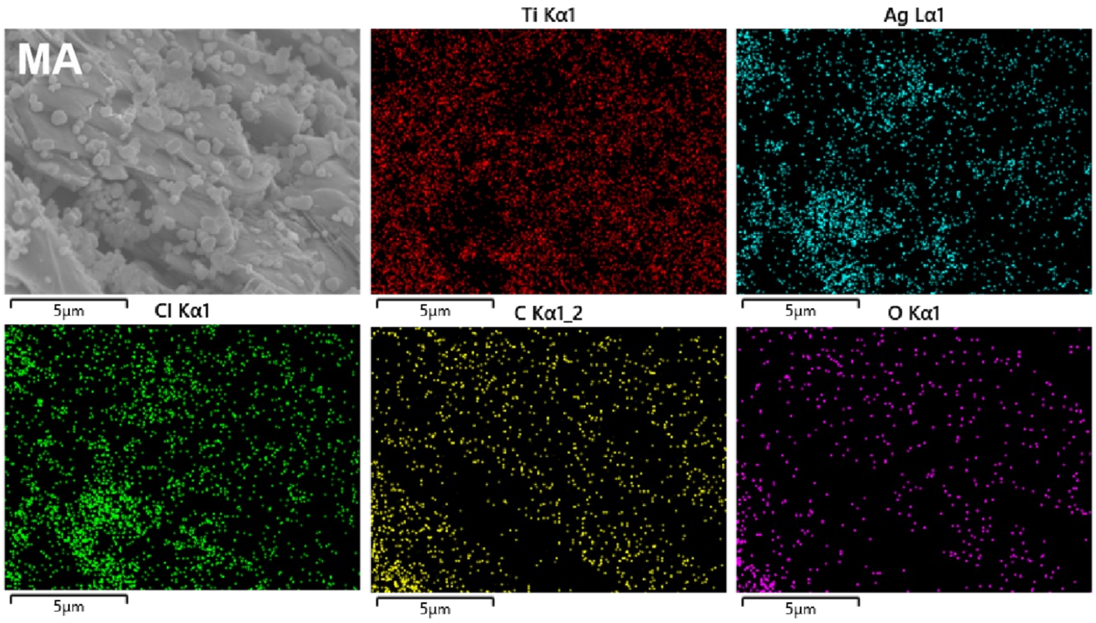

Fig. S1. The SEM images of MA filler and their mapping elements.

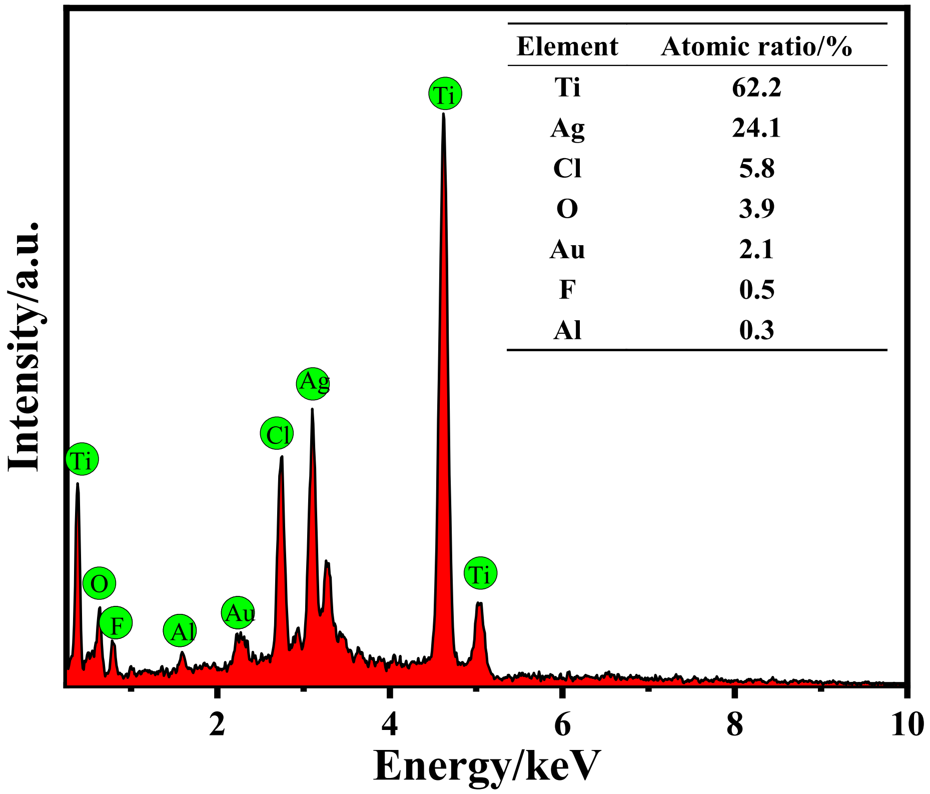

Fig. S2. The element mass proportions of MA filler from their element mappings.

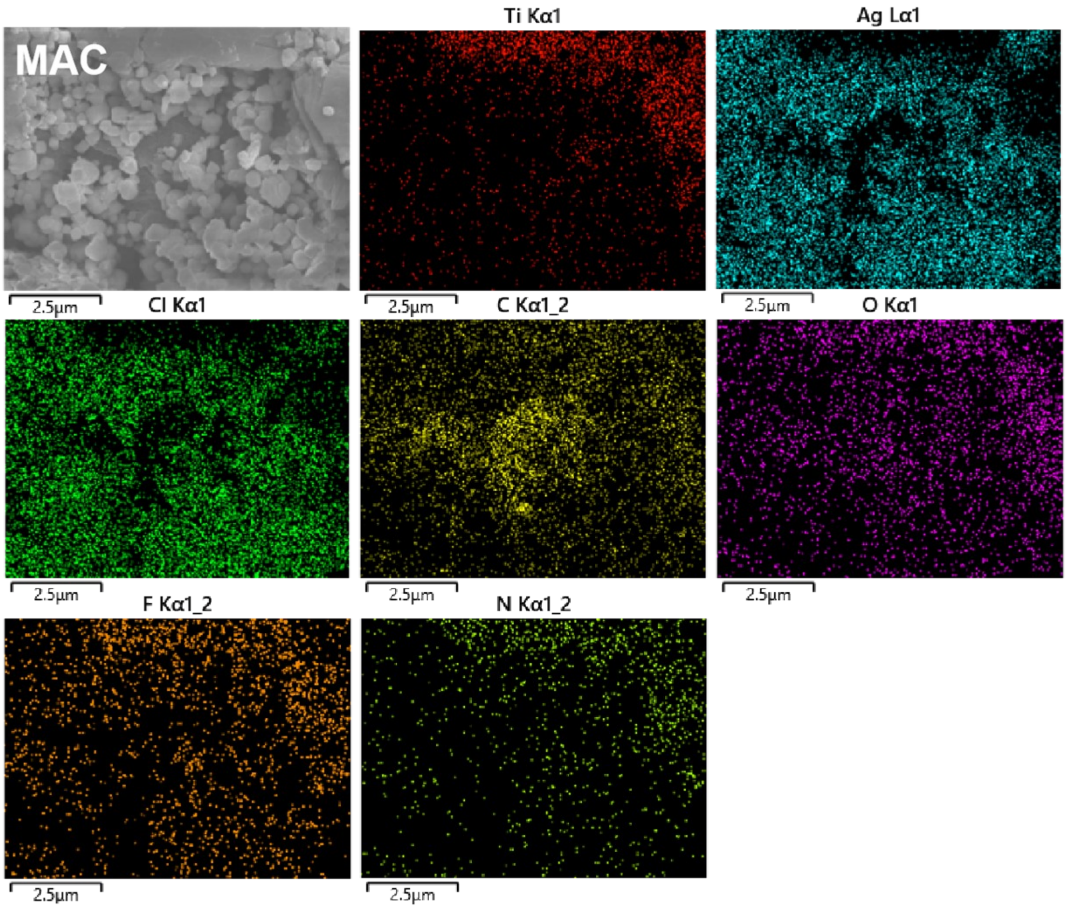

Fig. S3. The SEM images of MAC filler and their mapping elements.

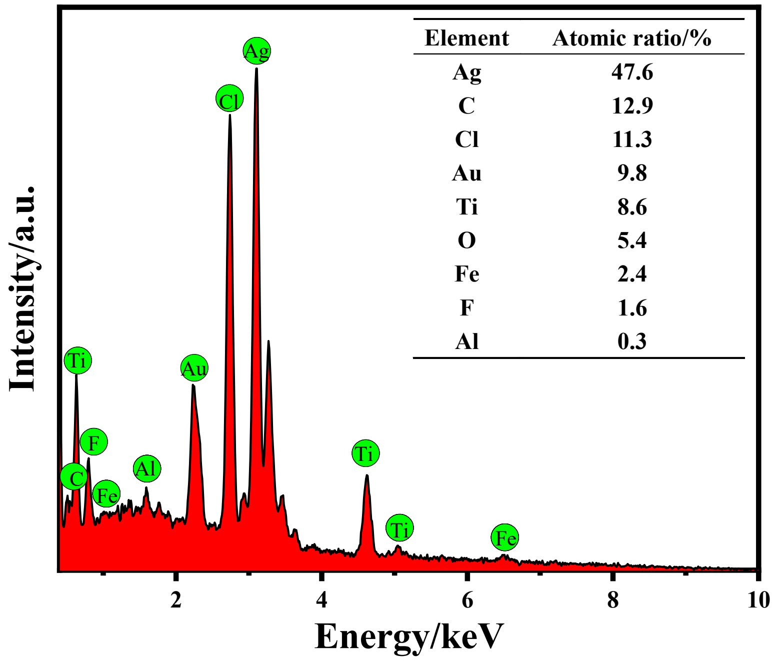

Fig. S4. The element mass proportions of MAC filler from their element mappings.

Because of the interference form the other substances such as the conductive tape on the sample table, we choose a small area of the hybrid fillers to analyze their elementary compositions, as shown in Figs. S1-4.
